# Full-Endoscopic Spinal Canal Decompression After Direct Midline Splitting of the Spinous Process: A Technical Note

**DOI:** 10.7759/cureus.89648

**Published:** 2025-08-08

**Authors:** Eitaro Okumura, Tatsuya Maegawa, Yosuke Nakayama, Hitoshi Hayase, Motoo Kubota

**Affiliations:** 1 Spinal Surgery, Kameda Medical Center, Chiba, JPN

**Keywords:** decompression laminectomy, full-endoscopic spine surgery, lumbar spinal canal stenosis, midline splitting of spinous process, minimally invasive surgical procedures

## Abstract

For lumbar spinal canal stenosis, endoscopic spine surgery typically employs a unilateral approach. While this approach has the advantage of early access to the lamina, it risks damage to the facet joint on the entry side. Additionally, decompression of the ipsilateral lateral recess can be challenging, sometimes resulting in inadequate decompression laterally, leading to incomplete symptom relief. To address these issues, a midline approach after midline splitting of the spinous process has been developed. However, this technique often uses a relatively large 16mm cylindrical retractor, resulting in skin incisions of approximately 20-25mm according to various reports. In our case, we performed a full-endoscopic spinal canal decompression underwater using a 7mm diameter through a 12mm skin incision, after directly splitting the spinous process along the midline. This technique achieved sufficient decompression with favorable outcomes. We report the details of this less invasive surgical procedure. The patient was a 62-year-old male who was independent in activities of daily living with a history of degenerative spondylolisthesis. This patient underwent posterior lumbar interbody fusion for L4/5 degenerative spondylolisthesis eight years ago. He had experienced pain in the left groin and perineal area (Numerical Rating Scale 6) for five months without improvement, which led him to our outpatient clinic. At the time of his visit, there was no apparent muscle weakness in either lower limb, only sensory disturbance. Lumbar MRI examination led to a diagnosis of lumbar spinal canal stenosis (L1/2) and conus medullaris syndrome. He requested endoscopic treatment, and we decided to perform an underwater full-endoscopic spinal canal decompression. The surgery involved a 12mm midline skin incision and direct splitting of the spinous process (L1) by approximately 10mm using a chisel and hammer. After placing a straight sheath, trumpet-shaped laminectomy was performed under endoscopic visualization. Trumpet-shaped refers to a laminectomy technique where the surgical field gradually widens as bone removal progresses deeper into the lamina. The yellow ligaments on both sides were removed as much as possible, resulting in sufficient decompression. Both facet joints were preserved, and extensive decompression was achieved. Postoperatively, although the patient still had some perineal pressure sensation, the sensory disturbance in the groin area improved, and he was discharged home on the second postoperative day with a modified Rankin Scale of 1. We report a case of successful spinal canal decompression using an endoscopic approach after midline splitting of the spinous process for lumbar spinal canal stenosis. We consider this surgical method to be valuable as it is less invasive, provides a good symmetrical view, and allows sufficient decompression on both sides.

## Introduction

Endoscopic decompression for lumbar spinal canal stenosis is minimally invasive with many advantages such as preservation of paraspinal muscles and supraspinous/interspinous ligaments, reduction in intraoperative blood loss, and minimization of bone removal. For bilateral decompression of lumbar spinal canal stenosis, the unilateral approach has been widely used [1,2]. While there is an advantage in early access to the lamina, there is a risk of damage to the facet joint on the entry side, and decompression of the ipsilateral lateral recess can be challenging, sometimes resulting in inadequate lateral decompression [3,4]. To overcome these issues, a midline approach after midline splitting of the spinous process has been developed. However, reports often describe using a 16mm cylindrical retractor, necessitating skin incisions of approximately 20-25mm [3,4]. In our case, aiming for further minimally invasive surgery, we performed a full-endoscopic spinal canal decompression underwater using a 7mm diameter through a 12mm skin incision after directly splitting the spinous process along the midline, achieving sufficient decompression with favorable outcomes. Since this surgical method can be a further minimally invasive laminectomy, we report it here.

## Technical report

The patient was a 62-year-old male who was independent in activities of daily living with a history of degenerative spondylolisthesis. This patient underwent posterior lumbar interbody fusion for L4/5 degenerative spondylolisthesis eight years ago. He had experienced pain in the left groin and perineal area (Numerical Rating Scale (NRS) 6) for five months without improvement, which led him to our outpatient clinic. At the time of his visit, there was no apparent muscle weakness in either lower limb, only sensory disturbance in the left groin and perineal area. Lumbar MRI examination led to a diagnosis of lumbar spinal canal stenosis (L1/2). He requested endoscopic treatment, and we decided to proceed with endoscopic laminectomy. We report the detailed surgical technique for this case.

From skin incision to endoscope insertion

The patient is positioned in the prone position under general anesthesia, similar to a standard laminectomy. Lower limb motor-evoked potential monitoring is used. A cushion is placed under the abdomen to create lordosis in the lumbar spine. After confirming under fluoroscopy that the lumbar spine is not rotated, the target spinous process is marked. A 12mm skin incision is made along the midline. The connective tissue and supraspinous ligament are incised along the midline using monopolar electrocautery, exposing the tip of the spinous process. The spinous process is split longitudinally by approximately 10mm using a double-edged chisel and hammer, then spread laterally to preserve the muscles attached to the spinous process.

Endoscopic procedure

**Figure 1:**
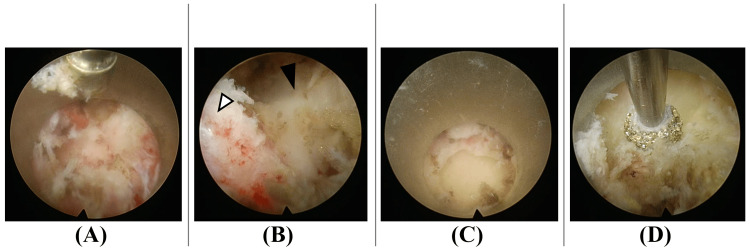
From endoscope insertion to resection of the spinous process base Upon initial endoscope insertion, the remaining spinous process in the midline is confirmed (A). While maintaining hemostasis, the spinous process (white arrowhead in panel B) and right lamina (black arrowhead in panel B) are identified. Drilling is completed to the base of the spinous process (C), and laminectomy is initiated from the midline (D).

A straight sheath is placed via a pencil dilator. Since the base of the spinous process remains thick, we use a 3.5mm diamond burr to thin the remaining spinous process while confirming the midline under frontal fluoroscopy (Figure 1). We use a Richard Wolff endoscope (Vernon Hills, IL, USA) with a 4.1mm working channel and 7mm outer diameter.

**Figure 2:**
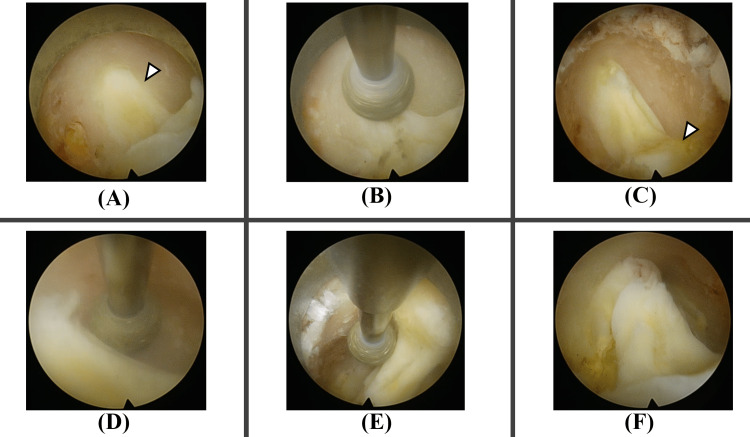
From laminectomy to before yellow ligament removal The yellow ligament in the midline area is exposed (white arrowhead in panel A). A symmetrical and good field of view is secured, making anatomical orientation easier. Drilling continues to expand the exposed area of the yellow ligament (B). The superficial layer of the yellow ligament continuing to the dorsal side of the lower lamina is confirmed (white arrowhead in panel C). Drilling continues toward the lateral recess with awareness of trumpet-shaped laminectomy (D). Drilling also continues caudally (E). The yellow ligament is sufficiently exposed (F).

Drilling continues toward the base of the spinous process until the yellow ligament becomes visible caudally. After confirming the position at the interspinous level and midline, bone removal is extended laterally in both directions and cranially (Figure 2).

**Figure 3:**
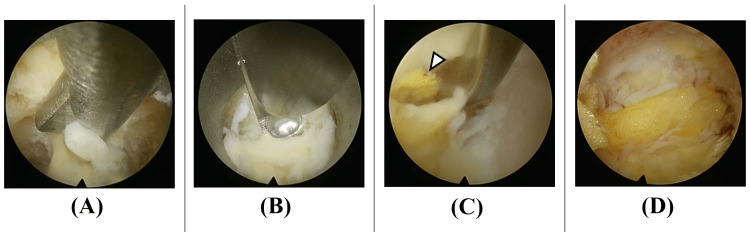
From yellow ligament detachment to confirmation of epidural fat layer The yellow ligament is removed using a dissector to confirm depth, along with basket forceps and Kerrison punch (A, B). When epidural fat appears (white arrowhead in panel C), proximity to the dural surface is recognized. Most of the yellow ligament has been removed, with epidural fat layer remaining (D).

After bone removal, the yellow ligament is removed as much as possible laterally on both sides, cranially up to the ligament attachment at the midline, and caudally to the ligament attachment on the lower lamina (Figure 3).

**Figure 4:**
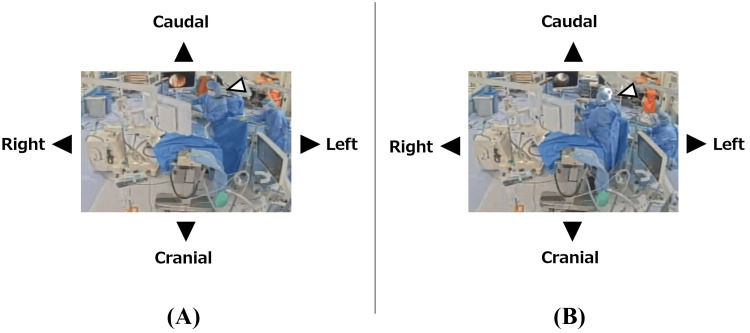
Surgeon's position The surgeon (white arrowhead in panels A and B) basically stands at the left caudal side of the prone patient and proceeds with the surgery while viewing the monitor on the right cranial side (A). When operating on the caudal part of the decompression area, the surgeon stands at the patient's left cranial side and proceeds with the surgery while viewing the monitor on the right caudal side (B).

The surgeon basically stands at the patient's left caudal side, but for caudal operations, the surgeon repositions to the patient's left cranial side (Figure 4).

**Figure 5:**
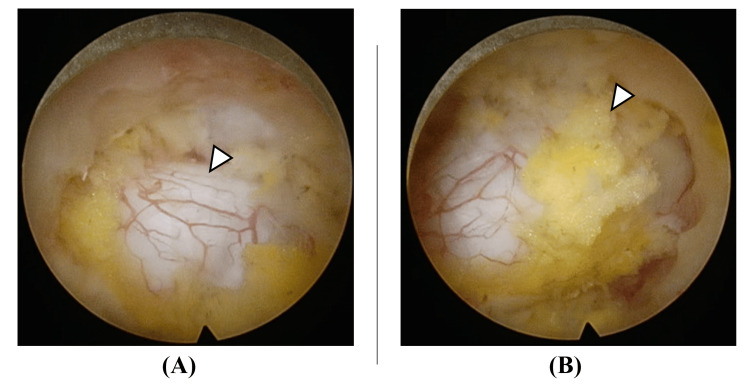
Final images Final image of the cranial area of decompression (A). The dural surface is exposed, and good dural pulsation can be confirmed (white arrowhead in panel A). Final image of the caudal area of decompression (B). Although epidural fat layer remains (white arrowhead in panel B), sufficient decompression has been achieved.

**Figure 6:**
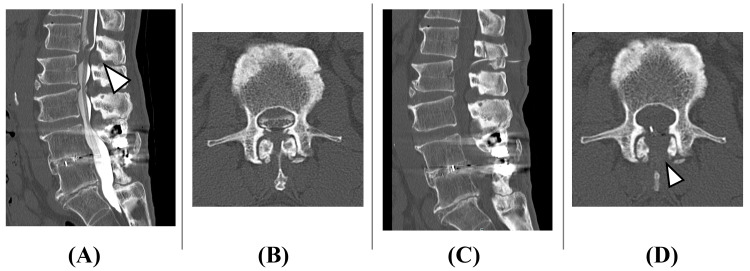
Lumbar CT images Preoperative lumbar sagittal section (A) and preoperative lumbar axial section (B) are CT images after myelography, showing severe spinal canal stenosis at the L1/2 level (white arrowhead in panel A). Postoperative lumbar sagittal section (C) and postoperative lumbar axial section (D) show that the spinous process has been split longitudinally and spinal canal decompression has been achieved. Despite the relatively narrow laminar width, both facet joints are preserved, and trumpet-shaped laminectomy has been performed (white arrowhead in panel D).

After circumferential removal of the yellow ligament, sufficient expansion of the spinal canal is achieved (Figures 5, 6).

Closure

After confirming hemostasis, a negative pressure drain is placed in the epidural space. The subcutaneous tissue and dermis are sutured with absorbable sutures.

Pain in the groin and perineal area began to improve from the first postoperative day. Three months after surgery, the groin pain had completely resolved (NRS 0), while some numbness in the perineal area persisted.

## Discussion

A systematic review and meta-analysis of 994 patients in 2019 concluded that endoscopic decompression for lumbar spinal canal stenosis resulted in statistically significant lower VAS scores for back and leg pain compared to microscopic decompression, with a 40% reduction in complication rates [5]. Reports of single-port endoscopic lumbar decompression are increasing, with many reporting satisfactory clinical results. Most of these single-port endoscopic lumbar decompression procedures use a unilateral approach with a paramedian skin incision of about 10mm and a 7mm outer diameter endoscopic system. Despite the notable advantages, preserving the facet joint can be difficult in cases with narrow laminae [1]. Particularly in elderly Asian patients with smaller vertebrae, minimizing bone removal may result in insufficient decompression, and the narrow field of view can make it difficult to judge anatomical orientation [6,7]. In contrast, the midline approach with splitting of the spinous process can overcome the issues of facet joint damage and difficulties in ipsilateral decompression encountered in the unilateral approach [8]. The midline approach is particularly significant for the upper lumbar spine, where the laminar width is narrower than in the lower lumbar spine. Additionally, there is no damage to the multifidus muscle, and bleeding from muscle layers is minimized. While there are reports of midline approaches using a 16mm cylindrical retractor [3,4], there are few reports of full-endoscopic underwater midline approaches using a 7mm outer diameter endoscopic system. Compared to conventional midline approaches, the skin incision is about half the size at 12mm, making it even less invasive. Additionally, since it is a completely underwater surgery, visibility of the surgical field is excellent. The view of both sides facilitates anatomical orientation and makes dissection of the yellow ligament easier. Furthermore, since this technique uses a 7mm outer diameter endoscopic system that is familiar to physicians involved in endoscopic spine surgery, reproducibility can be easily achieved once surgeons become oriented to laminectomy procedures. One issue is the longer operation time. Using only a 3mm drill for bilateral laminectomy is time-consuming. Mikami et al. reported that the average operation time for 10 cases of midline approach with a 16mm cylindrical retractor for lumbar spinal canal stenosis was 134.4 minutes (range 83-225 minutes), with an average blood loss of 70.7g (range 5-230g) [3]. In our case, the operation time was 178 minutes with a blood loss of 10ml. Accumulating cases to reduce operation time is a future challenge. Also, while the multifidus muscle is preserved, the spinous process and supraspinous ligament are partially damaged. The effect of damage to these posterior supporting structures, which contribute to spinal stability, also needs to be investigated in the future. Furthermore, endoscopes with a wider working channel diameter of 6.4mm, rather than 4.1mm, are also available for use [9,10]. Although the skin incision is slightly larger, the procedure is still performed completely underwater, and the advantage of minimal damage to surrounding structures such as muscles, facet joints, and interspinous ligaments remains the same. Takebayashi et al. conducted a comparative study of surgical outcomes between procedures using a 16mm tubular retractor and those using an endoscope with a 6.4mm working channel for single-level lumbar spinal stenosis, and reported that the surgical outcomes with the 6.4mm diameter endoscope were not inferior to those achieved with the conventional 16mm tubular retractor [11]. While the high-speed drill used with the 4.1mm working channel endoscope has a diameter of 3.5mm, the 6.4mm working channel endoscope allows the use of a 4.0mm diameter high-speed drill, which may contribute to reducing operative time.

## Conclusions

We report a case of successful spinal canal decompression using an endoscopic approach after midline splitting of the spinous process for lumbar spinal canal stenosis. This surgery using a 7mm endoscopic system is even less invasive than previously reported cases, providing a good field of view while achieving sufficient decompression on both sides. Although the longer operation time remains a challenge for the future, since this is only a single case report, further accumulation of cases is needed. It is desirable to continue research by accumulating cases and comparing recovery time, complications, patient satisfaction, and application to other spinal levels with conventional endoscopic laminectomy.
